# Dietary Mannan Oligosaccharide Supplementation During Gestation Alters Milk Composition in Jennies and Improves Growth Performance, Lipid Metabolism, and Gut Microbiota in Dezhou Donkey Foals

**DOI:** 10.3390/microorganisms14102409

**Published:** 2026-10-21

**Authors:** Huiying Li, Jia Luo, Zhangxinhan Wen, Yuhan Yin, Shimeng Huang, Donghui Shi

**Affiliations:** 1 College of Animal Husbandry and Veterinary Medicine, Jinzhou Medical University, Jinzhou 121001, China; 2 National Key Laboratory of Livestock and Poultry Nutrition and Feeding, College of Animal Science and Technology, China Agricultural University, Beijing 100193, China; 3 Laboratory of Feed Grain Safety and Healthy Poultry Farming, Beijing Jingwa Agricultural Science and Technology Innovation Center, Beijing 101206, China

**Keywords:** mannan oligosaccharides, Dezhou donkey, milk composition, host metabolism, gut microbiota, maternal nutrition

## Abstract

Mannan oligosaccharides (MOSs) are used as prebiotic feed additives, but their effects on lactating donkeys and their offspring remain poorly understood. This study evaluated the effects of maternal dietary MOS supplementation on milk composition, foal growth and serum biochemical parameters, and the fecal microbiota of Dezhou donkey foals. We randomly allocated 16 healthy lactating Dezhou donkeys and their foals to the control group (OCON) or the mannan-oligosaccharide supplementation group (OMOS; mannan-oligosaccharides were mixed into concentrate at 0.5 g/kg DM), with 8 replicates per group. The experiment lasted for 60 days. Maternal MOS supplementation did not significantly alter milk fat, protein, lactose, somatic cell count, or milk urea nitrogen, although milk fat and protein were numerically higher in the OMOS group; however, milk somatic cell count was significantly reduced in the OMOS group (*p* = 0.023). Foals from MOS-supplemented jennies tended to show greater total body weight gain and average daily gain. Serum total cholesterol and LDL-C concentrations were significantly lower, whereas HDL-C was significantly higher, in OMOS foals. MOS supplementation was associated with changes in specific bacterial taxa in the fecal microbiota of donkey foals, rather than a generalized alteration of the overall microbial community. Overall, maternal MOS supplementation was associated with favorable changes in foal growth and cholesterol metabolism, a reduction in milk somatic cell count, and selective alterations in fecal bacterial taxa.

## 1. Introduction

Mannan oligosaccharides (MOS) are non-digestible prebiotics primarily derived from the cell wall of Saccharomyces cerevisiae. Their characteristic α-1,2-, α-1,3-, and α-1,6-linked mannan structures confer relatively high stability during feed processing and passage through the gastrointestinal tract [1,2]. Previous studies have demonstrated that dietary MOS can modulate intestinal microbial communities, enhance immune function and antioxidant capacity, and influence host energy and lipid metabolism, supporting its potential use as an alternative to antibiotic growth promoters in animal production [3,4,5,6]. In weaned piglets, MOS supplementation has been reported to increase the CD4^+^/CD8^+^ ratio and average daily gain, reduce diarrhea incidence, and improve intestinal morphology and epithelial barrier function [3]. MOS may also exert immunomodulatory effects by suppressing pro-inflammatory cytokines, including IL-1β, IL-6, and IL-8, and regulating inflammation-related signaling pathways. For example, konjac-derived MOS have been shown to alleviate intestinal inflammation through macrophage SIGNR1-mediated signaling [6]. MOS are a class of functional oligosaccharides with potential prebiotic and other bioactive properties. Previous studies have shown that the biological effects of MOS may differ among animal species and experimental conditions [1]. Despite extensive research in pigs, poultry, and ruminants, information regarding the effects of MOS in lactating donkeys remains limited, particularly with respect to mammary secretion, milk composition, and milk metabolic profiles [7].

The Dezhou donkey is an important indigenous donkey breed in China and is widely raised in Shandong Province because of its large body size, adaptability to roughage-based diets, and economic value for hide, meat, and milk production [8]. Donkey milk shares several compositional characteristics with human milk, including relatively high lactose concentrations and a favorable protein and fatty acid profile, which has contributed to increasing interest in its nutritional and functional properties [9]. With the continued development of the donkey milk and ejiao industries, there is growing interest in nutritional strategies that can support lactation performance and offspring health [10]. The increasing emphasis on reducing antibiotic use in animal production has further stimulated interest in safe and sustainable feed-based alternatives [11]. Lactation imposes substantial metabolic and nutritional demands on jennies, and inadequate adaptation to these demands may impair nutrient utilization and mammary synthetic capacity [12]. Previous research on MOS supplementation in Dezhou donkeys has primarily focused on maternal circulating metabolites and offspring intestinal microbiota [7]. However, whether MOS supplementation alters conventional milk composition and endogenous milk metabolites, and how these changes are related to maternal systemic metabolism, remain poorly understood.

Recent studies have increasingly applied molecular and microbial approaches to investigate nutritional regulation in donkeys. For example, 16S rRNA gene sequencing has been used to characterize the effects of dietary interventions on hindgut microbial composition in growing donkeys [13], while transcriptomic studies have identified candidate genes associated with economically important traits such as collagen deposition in donkey skin [14]. However, relatively limited information is available regarding the effects of functional feed additives on lactating jennies and their offspring. A previous study evaluating dietary MOS supplementation in Dezhou donkeys reported changes in maternal metabolic characteristics and the fecal microbiota of foals [7]. Studies in lactating ruminants have further suggested that prebiotic supplementation may influence nutrient utilization, milk composition, and lipid metabolism [15,16]. Whether similar responses occur in lactating equids and whether maternal MOS supplementation can influence offspring growth and intestinal microbial characteristics remain unclear.

Therefore, the present study evaluated the effects of dietary MOS supplementation at 0.5 g/kg dry matter in lactating Dezhou jennies on milk composition, offspring growth performance and serum biochemical characteristics, and the fecal microbiota of donkey foals. We hypothesized that maternal dietary MOS supplementation could influence milk characteristics and offspring physiological responses, particularly growth, lipid metabolism, and fecal microbial composition. By integrating maternal milk characteristics with offspring phenotypic and microbiota data, this study aimed to provide further insight into the potential maternal–offspring effects of MOS supplementation and to provide a scientific basis for its nutritional application in lactating Dezhou jennies.

## 2. Materials and Methods

### 2.1. Ethics Statement

The animal study protocol was approved by the Animal Care and Use Committee of China Agricultural University (Approval No. AW90605202-1-05; approval date: 9 June 2025). All experimental procedures were conducted in accordance with institutional and national guidelines for the care and use of experimental animals, with appropriate measures taken to safeguard animal welfare throughout the study.

### 2.2. Experimental Design and Diets

Sixteen healthy lactating Dezhou jennies (2–3 years of age; body weight, 269.88 ± 31.84 kg) and their corresponding foals were enrolled in the experiment at the Dezhou Donkey Breeding Base of Shandong Dong’e Ejiao Co., Ltd. (Shandong, China). Jennies and their foals were housed together in semi-open pens under standardized management conditions. The jennies were fed a farm-formulated basal diet twice daily at 08:00 and 18:00 and had free access to clean drinking water. Foals were allowed to suckle their dams freely throughout the experimental period. Neither the jennies nor the foals received probiotics or antibiotics for at least 3 months before or during the experiment. Following a 3-day adaptation period, the 16 jenny–foal pairs were randomly allocated to two groups, with eight pairs per group. Jennies in the control group (OCON) received the basal diet alone, whereas those in the MOS-supplemented group (OMOS) received the same basal diet supplemented with 0.5 g/kg dry matter of mannan oligosaccharide (Actigen™, Beijing Alltech Biological Products Co., Ltd., Beijing, China). The formal experimental period lasted 60 days. The composition and calculated nutrient levels of the basal concentrate on a dry-matter basis are presented in Table S1.

### 2.3. Milk Composition Analysis

Milk fat, protein, lactose, somatic cell count (SCC), and milk urea nitrogen (MUN) were determined using a Lacticheck series automated milk analyzer (LC-01/LC-02, Tenovo, China) according to the manufacturer’s instructions. Each treatment group consisted of eight biological replicates, with one milk sample collected from each jenny for analysis.

### 2.4. Serum Biochemical Analysis

Serum total protein (TP; A045-2-2), albumin (ALB; A028-2-1), alanine aminotransferase (ALT; C009-2-1), aspartate aminotransferase (AST; C010-2-1), alkaline phosphatase (ALP; A059-2-2), total cholesterol (TC; A111-1-1), triglycerides (TG; A110-1-1), high-density lipoprotein cholesterol (HDL-C; A112-1-1), and low-density lipoprotein cholesterol (LDL-C; A113-1-1) were determined using commercially available assay kits (Nanjing Jiancheng Bioengineering Institute, Nanjing, China) according to the manufacturer’s instructions. Absorbance was measured using a spectrophotometer (Thermo Fisher Scientific, Waltham, MA, USA), and the concentrations or activities of the corresponding biochemical parameters were calculated according to the procedures specified for each assay kit. Each treatment group consisted of eight biological replicates, and each sample was analyzed in duplicate.

### 2.5. Fecal Microbiota Sequencing and Bioinformatics Analysis

Total microbial genomic DNA was extracted from approximately 200 mg of each fecal sample using the E.Z.N.A.^®^ Soil DNA Kit (Omega Bio-tek, Norcross, GA, USA) according to the manufacturer’s instructions. The V3–V4 hypervariable regions of the bacterial 16S rRNA gene were amplified using the universal primers 338F and 806R. PCR products were examined by 2% agarose gel electrophoresis, purified using the AxyPrep DNA Gel Extraction Kit (Axygen Biosciences, Union City, CA, USA), and quantified using a Qubit 2.0 Fluorometer (Thermo Fisher Scientific, Waltham, MA, USA). Equimolar amounts of purified amplicons were pooled and subjected to paired-end sequencing (2 × 250 bp) on an Illumina MiSeq platform (Illumina, San Diego, CA, USA) by Majorbio Bio-Pharm Technology Co., Ltd. (Shanghai, China).Raw sequencing reads were subjected to quality filtering, denoising, merging, and chimera removal using QIIME 2. Amplicon sequence variants (ASVs) were generated using the DADA2 algorithm. Taxonomic classification was performed against the SILVA reference database (release 138) using the RDP Classifier. Alpha diversity was evaluated using the ACE, Chao1, Shannon, and Sobs indices. Venn diagrams were generated to visualize shared and unique taxa between the OCON and OMOS groups at the ASV, phylum, and genus levels. Beta diversity was assessed by principal coordinate analysis (PCoA) based on Bray–Curtis dissimilarity, and differences in overall microbial community structure between groups were evaluated by permutational multivariate analysis of variance (PERMANOVA). The relative abundances of bacterial taxa were summarized at the phylum and genus levels. Differentially enriched taxa between the two groups were identified using linear discriminant analysis effect size (LEfSe), with an LDA score > 2.0 and *p* < 0.05 considered as the screening criteria.

### 2.6. Statistical Analysis

Each jenny–foal pair represented one experimental pairing, with eight pairs per treatment group. Maternal and offspring variables were analyzed separately according to the corresponding individual animal, while the correspondence between each jenny and her foal was considered when interpreting relationships among maternal metabolic parameters, milk composition, and offspring phenotypes. Data are presented as the mean ± standard deviation (SD). Statistical analyses of growth performance, serum biochemical parameters, and milk composition were performed using GraphPad Prism version 9.5 (GraphPad Software, San Diego, CA, USA). Differences between the OCON and OMOS groups were evaluated using a two-tailed unpaired Student’s *t*-test. Statistical significance was defined as *p* < 0.05, whereas 0.05 ≤ *p* < 0.10 was considered to indicate a tendency. Microbial community analyses were performed as described above, and LEfSe analysis was conducted using an LDA score threshold of >2.0 and *p* < 0.05.

## 3. Results

### 3.1. Effects of Maternal MOS Supplementation on Growth Performance of Donkey Foals

The effects of maternal dietary MOS supplementation on the growth performance of donkey foals are presented in Table 1. Initial body weight did not differ between the OCON and OMOS groups (*p* = 0.512). After the 60-day experimental period, maternal supplementation with mannan oligosaccharides (MOS) did not significantly affect the final body weight of the donkey foals (*p* = 0.133). Compared with the OCON group, the OMOS group showed an increasing trend in total body weight gain (31.75 vs. 25.63 kg; *p* = 0.091) and average daily gain (0.53 vs. 0.43 kg/day; *p* = 0.091).

### 3.2. Effects of Maternal MOS Supplementation on Serum Biochemical and Lipid Metabolism Parameters of Donkey Foals

Table 2 presents the effects of maternal dietary MOS supplementation on serum biochemical and lipid metabolism parameters in donkey foals. No statistically significant differences were observed between the OCON and OMOS groups in serum TP, ALB, ALT, AST, or ALP concentrations (*p* > 0.05). Compared with the OCON group, foals in the OMOS group had lower serum TC concentrations (2.00 vs. 2.35 mmol/L; *p* = 0.015) and LDL-C concentrations (0.40 vs. 0.48 mmol/L; *p* = 0.045), whereas HDL-C concentrations were higher (1.56 vs. 1.27 mmol/L; *p* = 0.002). No statistically significant difference was observed in serum TG concentrations between the two groups (*p* = 0.298). Because these serum biochemical and lipid variables were not prespecified as primary endpoints and multiple variables were evaluated, the observed differences should be interpreted as exploratory findings.

### 3.3. Effects of Dietary MOS Supplementation on Milk Composition of Lactating Dezhou Jennies

The effects of dietary MOS supplementation on the milk composition of lactating Dezhou jennies are presented in Table 3. No significant differences were observed between the OCON and OMOS groups in milk fat, milk protein, lactose, somatic cell count (SCC), or milk urea nitrogen (MUN) (*p* > 0.05). Milk fat and milk protein contents were numerically higher in the OMOS group than in the OCON group (0.65% vs. 0.87% and 1.69% vs. 1.79%, respectively), although these differences were not statistically significant (*p* = 0.134 and *p* = 0.157, respectively). Lactose content also did not differ significantly between the two groups (*p* = 0.224). In contrast, maternal dietary MOS supplementation significantly reduced SCC, from 11.88 × 10^3^ cells/mL in the OCON group to 6.38 × 10^3^ cells/mL in the OMOS group (*p* = 0.023). No significant difference was observed in MUN concentration between the two groups (20.26 vs. 21.55 mg/dL; *p* = 0.474).

### 3.4. Effects of Maternal MOS Supplementation on the Diversity and Community Structure of Fecal Microbiota in Donkey Foals

**Figure 1:**
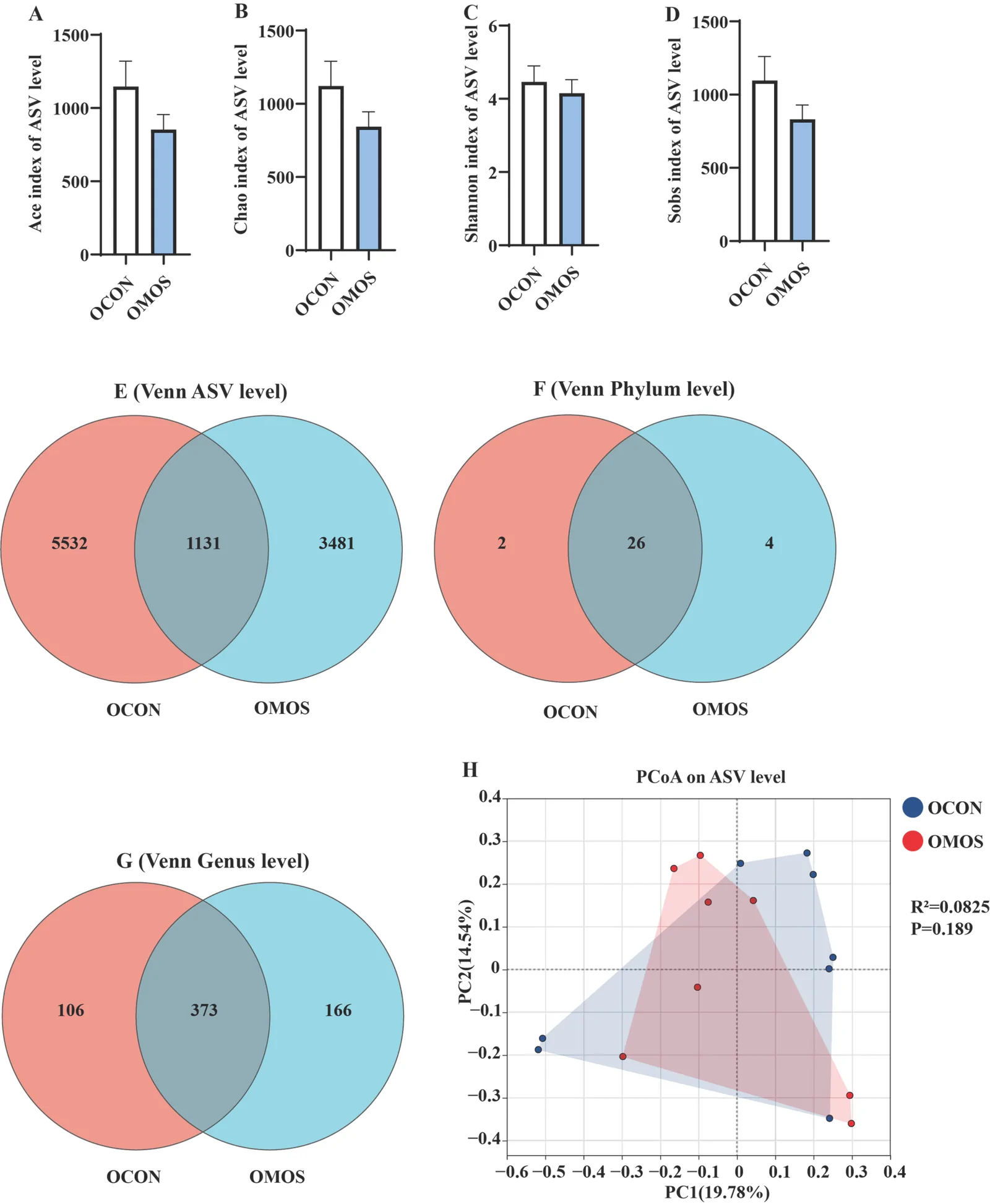
Effects of maternal dietary mannan oligosaccharide supplementation on fecal microbial diversity and community structure in Dezhou donkey foals. (**A**) ACE index; (**B**) Chao1 index; (**C**) Shannon index; (**D**) Sobs index; (**E**) Venn diagram at the ASV level; (**F**) Venn diagram at the phylum level; (**G**) Venn diagram at the genus level; and (**H**) principal coordinate analysis (PCoA) based on Bray–Curtis dissimilarity (PERMANOVA: R^2^ = 0.0825, *p* = 0.189). Data in panels (**A**–**D**) are presented as the mean ± SD (*n* = 8 per group).

The effects of maternal dietary MOS supplementation on the diversity and community structure of the fecal microbiota in donkey foals are shown in Figure 1. Alpha diversity was assessed using the ACE, Chao1, Shannon, and Sobs indices (Figure 1A–D). The ACE, Chao1, and Sobs indices, which primarily reflect microbial richness, as well as the Shannon index, which reflects overall community diversity, were numerically lower in the OMOS group than in the OCON group; however, none of these differences reached statistical significance (*p* > 0.05). These results indicated that maternal dietary MOS supplementation did not significantly affect the alpha diversity of the fecal microbiota in donkey foals. Venn diagrams were used to characterize shared and unique microbial taxa between the OCON and OMOS groups at the ASV, phylum, and genus levels (Figure 1E–G). At the ASV level, 1131 ASVs were shared between the two groups, whereas 5532 and 3481 ASVs were unique to the OCON and OMOS groups, respectively. At the phylum level, 26 phyla were shared between the two groups, with 2 phyla unique to the OCON group and 4 unique to the OMOS group. At the genus level, 373 genera were shared between the two groups, whereas 106 and 166 genera were unique to the OCON and OMOS groups, respectively. Principal coordinate analysis (PCoA) based on Bray–Curtis dissimilarity was performed to assess differences in the overall fecal microbial community structure between the two groups (Figure 1H). The samples from the OCON and OMOS groups showed partial overlap and separation in the PCoA ordination. PERMANOVA analysis indicated that treatment accounted for 8.25% of the variation in microbial community composition (R^2^ = 0.0825); however, the overall difference between the two groups was not statistically significant (*p* = 0.189). Collectively, maternal dietary MOS supplementation did not significantly alter either the alpha diversity or the overall community structure of the fecal microbiota in donkey foals.

### 3.5. Effects of Maternal MOS Supplementation on the Taxonomic Composition of Fecal Microbiota in Donkey Foals

**Figure 2:**
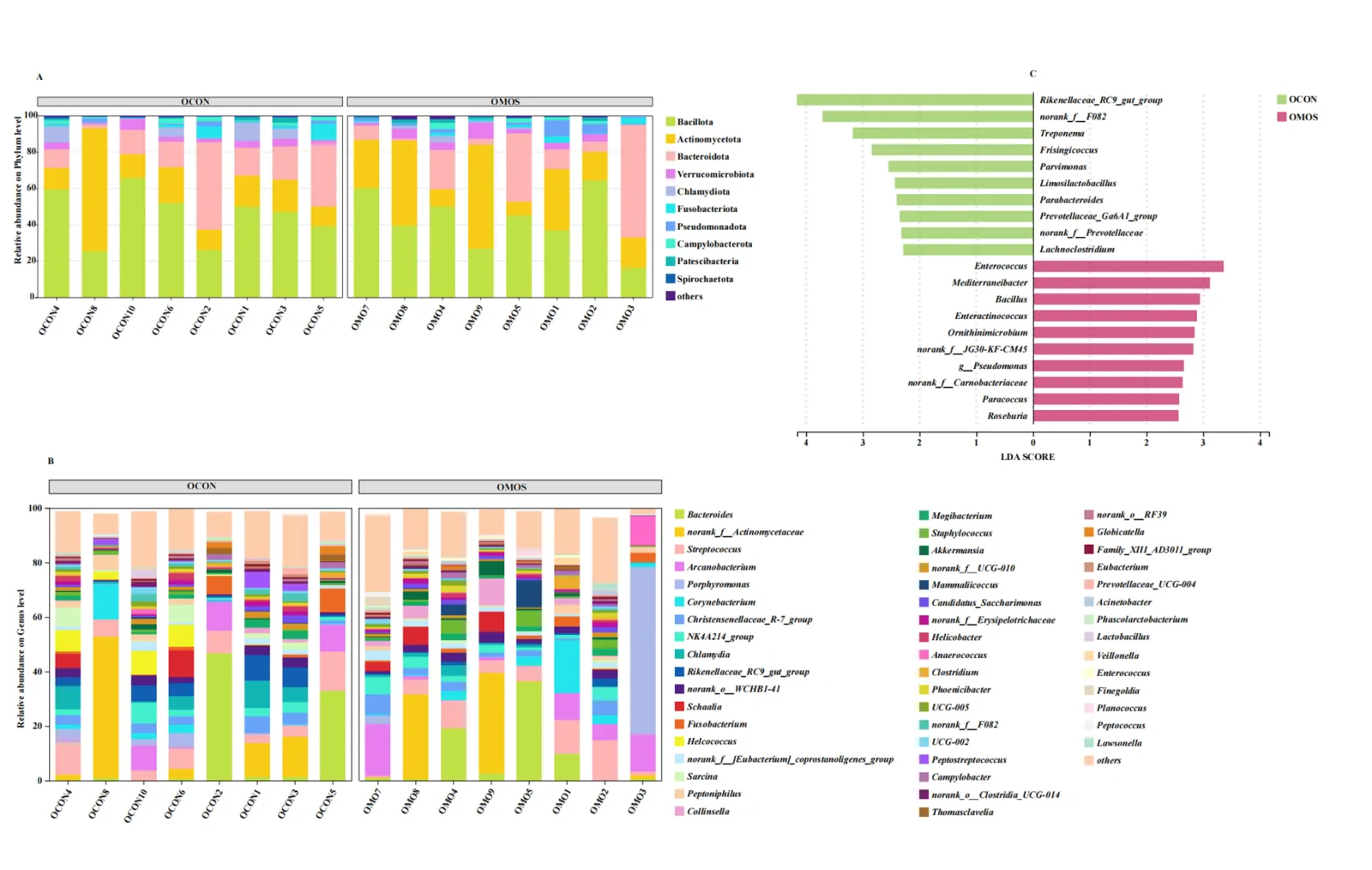
Effects of maternal dietary mannan oligosaccharide supplementation on fecal microbial composition and differential taxa in Dezhou donkey foals. (**A**) Relative abundance of the predominant bacterial phyla; (**B**) relative abundance of the predominant bacterial genera; and (**C**) linear discriminant analysis effect size (LEfSe) analysis showing differentially enriched bacterial taxa between the OCON and OMOS groups with an LDA score > 2.0. Green bars indicate taxa enriched in the OCON group, whereas pink bars indicate taxa enriched in the OMOS group. Each group contained eight biological replicates.

The taxonomic composition of the fecal microbiota in donkey foals was further characterized at the phylum and genus levels (Figure 2A,B). At the phylum level, Bacillota, Actinomycetota, and Bacteroidota were among the predominant phyla in both the OCON and OMOS groups (Figure 2A). The relative abundances of these major phyla varied among individual animals, whereas the remaining phyla, including Verrucomicrobiota, Chlamydiota, Fusobacteriota, Pseudomonadota, Campylobacterota, Patescibacteria, and Spirochaetota, generally represented smaller proportions of the fecal microbial community. At the genus level, the fecal microbiota exhibited considerable inter-individual variation in both groups (Figure 2B). Multiple genera contributed to the overall microbial community, with differences in their relative abundance profiles observed between individual foals and between the two treatment groups.

LEfSe analysis was subsequently performed to identify taxa differentially enriched between the OCON and OMOS groups (Figure 2C; LDA score > 2.0). The OCON group was characterized by greater enrichment of Rikenellaceae_RC9_gut_group, norank_f__F082, Treponema, Frisingicoccus, Parvimonas, Limosilactobacillus, Parabacteroides, Prevotellaceae_Ga6A1_group, norank_f__Prevotellaceae, and Lachnoclostridium. In contrast, Enterococcus, Mediterraneibacter, Bacillus, Enteractinococcus, Ornithinimicrobium, norank_f__JG30-KF-CM45, g__Pseudomonas, norank_f__Carnobacteriaceae, Paracoccus, and Roseburia were enriched in the OMOS group. These results indicate that, although maternal MOS supplementation did not significantly alter the overall microbial community structure as shown by PCoA and PERMANOVA analysis, it was associated with differential enrichment of specific bacterial taxa in the fecal microbiota of donkey foals.

## 4. Discussion

The present study evaluated whether maternal dietary supplementation with MOS during lactation could influence milk composition and, consequently, early growth, circulating metabolic status, and fecal microbial characteristics in Dezhou donkey foals. The major findings were that maternal MOS supplementation was associated with numerical increases in milk fat and protein contents, which were 0.65% vs. 0.87% and 1.69% vs. 1.79% in the OCON and OMOS groups, respectively, corresponding to numerical increases of 33.85% and 5.92%; however, these differences did not reach statistical significance (*p* = 0.134 and 0.157, respectively). In contrast, milk somatic cell count was significantly reduced by 46.21% in the OMOS group (*p* = 0.023). These findings suggest that MOS supplementation may have influenced certain aspects of milk composition, particularly milk fat and protein, although the evidence for these changes remains limited given the lack of statistical significance. The reduction in somatic cell count may indicate a potential improvement in mammary inflammatory status; however, this interpretation should be made cautiously because additional indicators of mammary health were not evaluated in the present study. In addition, maternal MOS supplementation did not significantly affect fecal microbial alpha diversity or overall community structure but was associated with differential enrichment of several bacterial taxa. Collectively, these findings suggest that maternal MOS supplementation exerts relatively subtle effects on conventional milk traits and the global fecal microbial community while being associated with more distinct changes in offspring lipid metabolism and specific microbial taxa.

Lactation is a physiologically demanding period characterized by substantial nutrient and energy requirements, and inadequate metabolic adaptation may compromise nutrient utilization and mammary secretory function in lactating animals [7,8,11]. In the present study, dietary supplementation with 0.5 g/kg MOS did not significantly alter milk fat, milk protein, lactose, somatic cell count, or milk urea nitrogen in lactating Dezhou jennies. Nevertheless, milk fat and protein contents were numerically higher in the OMOS group than in the OCON group, suggesting that MOS supplementation may have modestly influenced mammary nutrient secretion under the present experimental conditions. Similar effects of prebiotic oligosaccharides on nutrient utilization and milk composition have been reported in lactating livestock [15,17]. However, because the increases in milk fat and protein did not reach statistical significance, these findings should be interpreted cautiously and require confirmation using larger sample sizes and multiple supplementation levels. Milk lipid and protein synthesis depends largely on the availability and uptake of circulating metabolic precursors by mammary epithelial cells [18,19]. As a non-digestible yeast-derived prebiotic, MOS may influence host nutrient metabolism indirectly through modulation of gastrointestinal microbial fermentation and the production of microbial metabolites such as short-chain fatty acids (SCFAs) [20,21]. These metabolites can participate in energy metabolism and may affect the supply of substrates available for mammary synthesis. In addition, the metabolomic changes observed in maternal plasma and milk in a previous study suggest that MOS supplementation may influence both systemic metabolism and the metabolic composition of donkey milk [7]. Nevertheless, the absence of significant changes in conventional milk composition indicates that the effects of MOS may be more readily reflected in specific metabolic pathways or bioactive milk components than in major milk nutrients.

Maternal nutrition and milk-derived nutrients and bioactive compounds are important determinants of early postnatal growth and metabolic development [22,]. In the present study, the initial body weights of foals were comparable between groups, and maternal MOS supplementation did not significantly affect final body weight after the 60-day experimental period. However, total body weight gain and average daily gain were approximately 24% higher in the OMOS group than in the OCON group, with both variables showing a statistical tendency. These findings suggest that maternal MOS supplementation may support early growth in suckling foals, although this effect should be interpreted cautiously given the modest sample size and relatively short experimental period. Because milk represents the primary nutritional source for young suckling foals [23], maternal dietary supplementation may influence offspring growth through changes in nutrient supply, milk-derived bioactive factors, or other maternal–offspring interactions. However, the present data do not allow these potential mechanisms to be distinguished. Maternal MOS supplementation also altered several serum lipid-related indices in the foals. Serum TP, ALB, ALT, AST, and ALP did not differ significantly between groups, indicating that the measured indices of protein metabolism and hepatic biochemical status remained generally comparable. In contrast, foals in the OMOS group had significantly lower TC and LDL-C concentrations and higher HDL-C concentrations, whereas TG remained unchanged. This selective alteration in cholesterol-related indices suggests that maternal MOS supplementation may have influenced cholesterol transport, distribution, or turnover rather than causing a generalized change in circulating lipid concentrations. Maternal nutritional interventions during early development have previously been associated with alterations in offspring lipid metabolism [24], and changes in the relative distribution of HDL-C and LDL-C can reflect modifications in cholesterol transport among tissues [25]. Accordingly, the present findings indicate that maternal MOS supplementation was associated with an altered lipid metabolic status in suckling donkey foals. The mechanisms underlying these changes cannot be directly determined from the present study. Gut microorganisms and their metabolites are increasingly recognized as important modulators of host lipid metabolism, particularly through pathways related to bile acid metabolism and other microbially derived metabolites [26,27]. Previous studies have also shown that maternal prebiotic supplementation can be associated with metabolic changes in offspring [28]. In addition, a previous metabolomic study in Dezhou donkeys reported that dietary MOS supplementation altered circulating metabolic pathways related to host metabolism [7]. These earlier findings provide biological context for the lipid-related responses observed in the present study but should not be interpreted as direct mechanistic evidence for the current experiment. Therefore, the observed changes in serum cholesterol-related indices are best interpreted as physiological associations with maternal MOS supplementation rather than evidence of a defined metabolite-mediated pathway.

The gastrointestinal microbiota represents another potential link between maternal nutrition and offspring metabolism. The equine hindgut contains a highly complex microbial ecosystem that contributes substantially to the fermentation of dietary substrates and production of microbial metabolites [29,30]. Early-life microbial colonization is particularly dynamic and can be influenced by maternal diet, milk composition, environmental exposure, housing conditions, and direct contact with the dam. In the present study, however, maternal MOS supplementation did not significantly affect the ACE, Chao1, Shannon, or Sobs indices in donkey foals. Thus, neither microbial richness nor overall alpha diversity was significantly altered. Similarly, PCoA based on Bray–Curtis dissimilarity showed only partial separation between the two groups, and PERMANOVA analysis confirmed that the overall treatment effect on microbial community structure was not statistically significant. These findings indicate that maternal MOS supplementation did not induce a major restructuring of the fecal microbial ecosystem during the experimental period. The Venn analysis nevertheless revealed differences in the numbers of unique microbial features between groups. At the ASV level, the OCON group contained more unique ASVs than the OMOS group, whereas at the genus level, more unique genera were detected in the OMOS group. These results illustrate differences in microbial membership but should not, by themselves, be interpreted as evidence of increased or decreased microbial diversity because Venn diagrams are strongly influenced by the detection of relatively low-abundance taxa. Consistent with the alpha- and beta-diversity analyses, the taxonomic composition at the phylum level was broadly similar between groups, with Bacillota, Actinomycetota, and Bacteroidota representing the predominant phyla. Considerable inter-individual variation was observed, which may also have contributed to the lack of a statistically significant treatment effect on overall microbial community structure. Despite the absence of global community-level differences, LEfSe analysis identified several taxa that were differentially enriched between treatments. Rikenellaceae_RC9_gut_group, norank_f__F082, *Treponema*, *Frisingicoccus*, *Parvimonas*, *Limosilactobacillus*, *Parabacteroides*, Prevotellaceae_Ga6A1_group, norank_f__Prevotellaceae, and *Lachnoclostridium* were enriched in the OCON group, whereas *Enterococcus*, *Mediterraneibacter*, *Bacillus*, *Enteractinococcus*, *Ornithinimicrobium*, norank_f__JG30-KF-CM45, *Pseudomonas*, norank_f__Carnobacteriaceae, *Paracoccus*, and *Roseburia* were enriched in the OMOS group. Several bacterial groups identified in the present study have previously been associated with carbohydrate fermentation, SCFA production, intestinal ecological interactions, and host metabolic regulation [18,31,,32]. However, these reported associations do not establish that the corresponding metabolic activities occurred in the present animals. In particular, taxonomic shifts in bacteria previously associated with carbohydrate fermentation or SCFA production may be relevant to microbial metabolite availability; however, their actual metabolic activity and SCFA production cannot be inferred from the present 16S rRNA sequencing data. This provides a biologically plausible framework through which relatively subtle taxonomic changes might occur alongside alterations in circulating cholesterol-related parameters even in the absence of marked differences in overall microbial diversity. However, the differential taxa identified here should be interpreted with caution. First, enrichment of individual bacterial genera cannot automatically be equated with a beneficial or detrimental effect because the metabolic functions of many taxa are species-, strain-, substrate-, and host-dependent. For example, although some members of *Bacillus* and *Roseburia* have been associated with fermentation and metabolite production, other differentially abundant genera may have highly context-dependent biological roles. Similarly, several taxa enriched in the OCON group, including members of Prevotellaceae and other fermentative bacteria, may also participate in carbohydrate degradation. Therefore, the present microbiota results are more appropriately interpreted as selective ecological shifts rather than a simple transition from an unfavorable to a favorable microbial community. Second, LEfSe identifies discriminatory taxa based on relative abundance and effect size but does not establish their metabolic activity or causal contribution to the host phenotype. Consequently, 16S rRNA gene sequencing alone is insufficient to determine whether the observed microbial changes directly contributed to the altered serum lipid profile. Moreover, neither fecal SCFA concentrations nor microbial functional pathways were measured in the present study, and therefore the potential link between microbial taxonomic shifts, SCFA production, and serum lipid changes remains untested.

Taken together, the simultaneous occurrence of a tendency toward improved growth, significant changes in circulating cholesterol-related parameters, and selective enrichment of fecal bacterial taxa raises the possibility of coordinated interactions among maternal nutrition, milk-derived factors, offspring intestinal microbiota, and host metabolism [22]. Nevertheless, the present findings provide stronger evidence for an association between maternal MOS supplementation and offspring cholesterol metabolism than for broad alterations in milk nutrient composition or overall fecal microbial community structure. Although several bacterial taxa showed differential enrichment, overall microbial diversity and community structure remained largely unchanged between groups, indicating that the microbiota response was characterized by selective compositional shifts rather than extensive community-wide remodeling. Furthermore, because microbial metabolites and functional genes were not directly assessed, these microbial changes should not be interpreted as direct evidence of a microbiota-mediated mechanism. Overall, maternal MOS supplementation appears to primarily affect lipid metabolic status in donkey foals, with accompanying modest growth responses and selective alterations in the fecal microbiota.

## 5. Conclusions

In conclusion, maternal dietary supplementation with 0.5 g/kg MOS for 60 days was associated with favorable growth and metabolic responses in Dezhou donkey foals. Maternal MOS supplementation tended to increase total body weight gain and average daily gain and significantly altered circulating cholesterol-related parameters, as evidenced by reduced serum TC and LDL-C concentrations and increased HDL-C concentrations. Although MOS supplementation did not significantly affect fecal microbial alpha diversity or overall community structure, it was associated with the selective enrichment of several bacterial taxa, including *Enterococcus*, *Mediterraneibacter*, *Bacillus*, *Paracoccus*, and *Roseburia*. Collectively, these findings suggest that maternal MOS supplementation may influence early growth and lipid metabolism in donkey foals, potentially through coordinated interactions among maternal nutritional status, milk-derived factors, and specific gut microbial taxa. These results support the potential application of MOS as a nutritional strategy for lactating Dezhou jennies and provide a basis for further investigation of the maternal milk–offspring axis underlying its effects on offspring metabolism and development.

## Figures and Tables

**Table 1:** Effects of maternal dietary mannan oligosaccharide supplementation on the growth performance of Dezhou donkey foals.

Group	Initial Body Weight (kg)	Final Body Weight (kg)	Total Body Weight Gain (kg)	Average Daily Gain (kg/day)
OCON	68.75 ± 8.31	94.38 ± 4.82	25.63 ± 6.09	0.43 ± 0.10
OMOS	66.56 ± 3.95	98.31 ± 5.05	31.75 ± 7.37	0.53 ± 0.12
*p*-value	0.512	0.133	0.091	0.091

Data are presented as the mean ± SD (*n* = 8 per group). Differences were considered statistically significant at *p* < 0.05, whereas 0.05 ≤ *p* < 0.10 was considered to indicate a tendency.

**Table 2:** Effects of maternal dietary mannan oligosaccharide supplementation on serum biochemical parameters in Dezhou donkey foals.

Group	TP (g/L)	ALB (g/L)	ALT (U/L)	AST (U/L)	ALP (U/L)	TC (mmol/L)	TG (mmol/L)	HDL-C (mmol/L)	LDL-C (mmol/L)
OCON	59.30 ± 3.97	28.20 ± 1.97	6.41 ± 1.29	225.26 ± 31.74	255.21 ± 61.78	2.35 ± 0.32 ^a^	0.44 ± 0.11	1.27 ± 0.09 ^b^	0.48 ± 0.10 ^a^
OMOS	62.13 ± 3.81	28.20 ± 1.57	7.07 ± 1.49	206.16 ± 30.40	269.30 ± 33.89	2.00 ± 0.13 ^b^	0.51 ± 0.16	1.56 ± 0.19 ^a^	0.40 ± 0.04 ^b^

Data are presented as the mean ± SD (*n* = 8 per group). Differences were considered statistically significant at *p* < 0.05, while 0.05 ≤ *p* < 0.10 was considered to indicate a statistical tendency. Abbreviations: TP, total protein; ALB, albumin; ALT, alanine aminotransferase; AST, aspartate aminotransferase; ALP, alkaline phosphatase; TC, total cholesterol; HDL-C, high-density lipoprotein cholesterol; LDL-C, low-density lipoprotein cholesterol.Different superscript letters (a, b) within the same row indicate significant differences between groups (*p* < 0.05).

**Table 3:** Effects of maternal dietary mannan oligosaccharide supplementation on milk nutrient composition in lactating Dezhou donkeys.

Group	Milk Fat Content/%	Milk Protein Content/%	Lactose Content/%	Somatic Cell Count/10^3^ Cells/mL	Milk Urea Nitrogen/mg/dL
OCON	0.65 ± 0.25	1.69 ± 0.14	6.95 ± 0.13	11.88 ± 6.13 ^a^	20.26 ± 3.58
OMOS	0.87 ± 0.26	1.79 ± 0.12	6.85 ± 0.17	6.38 ± 3.07 ^b^	21.55 ± 2.92
*p*-value	0.134	0.157	0.224	0.023	0.474

Data are presented as the mean ± SD (*n* = 8 per group). Differences were considered statistically significant at *p* < 0.05, whereas 0.05 ≤ *p* < 0.10 was considered to indicate a tendency.

## Data Availability

The original contributions presented in this study are included in the article/Supplementary Material. Further inquiries can be directed to the corresponding authors.

## Supplementary Materials

The following supporting information can be downloaded at: https://www.mdpi.com/article/10.3390/microorganisms14102409/s1. Table S1: Composition and nutritional level of experimental concentrate (dry-matter basis).
