# Supplementary material for: Dietary Mannan Oligosaccharide Supplementation During Gestation Alters Milk Composition in Jennies and Improves Growth Performance, Lipid Metabolism, and Gut Microbiota in Dezhou Donkey Foals
Source: Microorganisms. 2026 Oct 21;14(10):2409. doi: 10.3390/microorganisms14102409 (PMC13707989; doi:10.3390/microorganisms14102409)
Supplement: Supplementary file 1 [file microorganisms-14-02409-s001.zip › microorganisms-4529821-supplementary.pdf]

Table S1. Composition and nutritional level of experimental concentrate (dry-matter basis).

Ingredients	Content (%)	Nutrients	Nutrient level (%)
Corn	31.75	Dry matter	88.53
Middlings bran	12.00	Crude protein	17.18
Wheat flour middling	21.00	Ash	8.37
DDGS	5.00	Crude fiber	4.27
Wheat bran	18.00	Ether extract	4.59
Soybean meal	6.65	Calcium	1.27
NaCl	0.63	Phosphorus	0.52
CaCO3	3.72	Lysine	0.75
CaHPO4	0.25		
Premix1	1.00		
Total	100.00		

[1] Premix/kg: VA, 600 KIU; VD3, 125 KIU; VE, 3500 IU; Fe, 2 g; Cu, 800 mg; Zn, 6 g; Mn, 13 g; I, 95 mg; Se, 50 mg.
